# The Spectrum of Shoulder Pathologies on Magnetic Resonance Imaging: A Pictorial Review

**DOI:** 10.7759/cureus.44801

**Published:** 2023-09-06

**Authors:** Anshul Sood, Shivali V Kashikar, Gaurav V Mishra, Pratapsingh Parihar, Shreya Khandelwal, Manasa Suryadevara, Nishtha Manuja, Keyur Saboo, Nitish Batra, Abhinav Ahuja

**Affiliations:** 1 Radiodiagnosis, Jawaharlal Nehru Medical College, Datta Meghe Institute of Higher Education and Research, Wardha, IND; 2 Medicine, Jawaharlal Nehru Medical College, Datta Meghe Institute of Higher Education and Research, Wardha, IND

**Keywords:** pathology, radiology, pictorial, shoulder, mri

## Abstract

Patients present to the orthopedic outpatient department with complaints of shoulder pain on movement or restriction of movement in the shoulder joint and are referred for magnetic resonance imaging (MRI) of the shoulder joint. Almost all the patients have similar complaints but may have a wide range of pathology affecting the joint and causing pain. Rotator cuff tears or tendinopathy are the most common causes of shoulder pain. Ultrasound (USG) and MRI are the most commonly used imaging modalities for assessing rotator cuff pathologies. There is a wide range of pathologies affecting the shoulder joint, other than rotator cuff tendinopathies or tears, for which USG is less sensitive and specific in detecting accurate pathology. MRI is the choice of imaging for shoulder joint pathologies. We present a pictorial review discussing and depicting MRI features of a wide list of pathologies of the shoulder joint complex that should be kept in mind when the patient presents with shoulder pain.

## Introduction and background

The shoulder complex consists of the acromioclavicular and glenohumeral joints. The latter is a type of synovial ball-and-socket joint involving the glenoid fossa of the scapula and the head of the humerus. It allows for a diversity of movements making it the most mobile and least stable joint of the human body. The acromioclavicular joint is a type of diarthrodial synovial joint and allows shoulder abduction and flexion. The shoulder joint comprises two dynamic stabilizers - biceps tendon and rotator cuff muscles; and three static stabilizers - glenoid labrum, capsular ligaments, and negative intra-articular pressure. Supraspinatus, subscapularis, infraspinatus, and teres minor make up the rotator cuff complex which holds the humeral head in the glenoid cavity. A musculotendinous collar encompasses the joint and provides stability, except in the inferior location [1].

One of the most common complaints involving the musculoskeletal system is pain in the shoulder region, with some studies showing an annual incidence of 14.7 per 1000 patients and a lifetime prevalence of 70% [2,3]. Internal disorders of the shoulder joint, including the pathologies of the joints, tendons, muscles, and periarticular and labroligamentous structures, are the frequent originators of pain in the shoulder region. Diagnostic imaging has gained importance in evaluating these structures and finding the cause of shoulder pain. With the development of surface coils used in shoulder joint imaging, MRI has become a crucial diagnostic tool having a high-spatial-resolution and multiplanar imaging capability for assessing the shoulder joint, surrounding ligaments, muscles, and soft tissues [4].

This pictorial essay will review the MRI appearance of certain disorders and pathologies of the rotator cuff muscles and tendons, acromioclavicular joint, labrum and glenohumeral joint, and the shoulder bursa. An informed verbal and written consent has been obtained from the patient.

## Review

Hemophilic arthropathy

**Figure 1:**
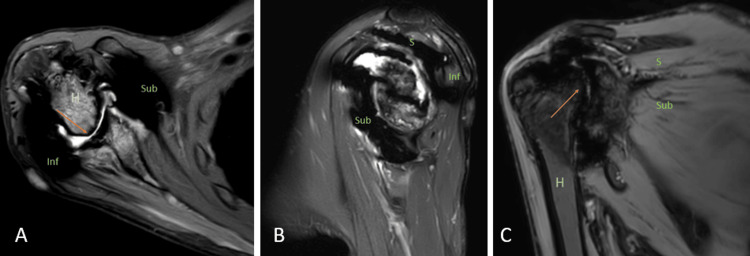
A 39-year-old male with hemophilic arthropathy. Axial fat-suppressed proton density (A), sagittal fat-suppressed proton density (B), and coronal susceptibility-weighted imaging (C) show erosive destruction of the head of humerus {H} with loss of contour and erosions of the visualized part including the greater and lesser tuberosity and glenoid margins (arrows) with hypo intensity of visualized subscapularis {Sub}, supraspinatus {S} and infraspinatus {Inf} on fat-suppressed proton density and blooming on susceptibility-weighted imaging. Image credits: Anshul Sood

Hemophilic arthropathy is a term for developing permanent joint disease as a long-term complication of hemarthrosis in hemophilia patients. Hemarthrosis can develop spontaneously or post-trauma, the shoulder being the least common joint, and is rarely seen in adults. The same joint can be affected repeatedly. Iron starts to deposit intraarticularly due to repeated hemarthrosis and leads to damage to the subchondral bone and articular cartilage [5,6]. MRI is the prime modality for detecting the early stages of the disease. Synovial thickening with low signal intensity because of the hemosiderin susceptibility effect is characteristic of siderotic synovitis. Other MRI features may include joint effusion, synovitis causing enhancement of synovium, and, ultimately, erosion and loss of cartilage (Figures 1A, 1B, 1C) [5].

Tuberculous arthropathy

*Mycobacterium tuberculosis* is the most common cause of tuberculosis (TB), with other rare causes including *Mycobacterium bovis* and other atypical mycobacteria [7]. Direct inoculation of TB affecting a musculoskeletal joint is infrequent and mainly caused by lymphatic or hematogenous spread from a primary focus of infection [8]. Sometimes, a dormant infection can get reactivated following an injury [9].

The initial findings of the TB of a joint include the formation of a bulky and congested synovial membrane with joint effusion. Later, cartilage destruction and bone erosion happen with the expansion of the granulomatous synovial lesions at the synovial reflections. Due to the lack of proteolytic enzymes in the exudates of tubercular arthritis, cartilage loss is often a late finding of the disease [10]. As the disease progresses, the osteolytic bony lesions develop [7]. If adequate treatment is not provided, the disease may form cold abscesses, periarticular soft tissue masses, and sinus tracts [10].

**Figure 2:**
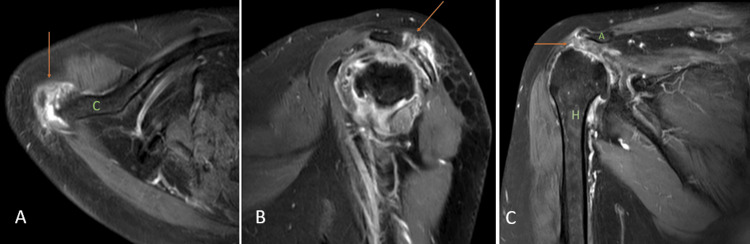
A 56-year-old female with tuberculous arthropathy. Contrast-enhanced T1 weighted axial (A), sagittal (B), and coronal (C) images show enhancing hyperintensities in the acromion {A} and distal clavicular {C} joint with decreased joint spaces and heterogeneously enhancing collection in the subacromial and subcoracoid bursa (arrows). Image credits: Anshul Sood

The role of USG in this pathology is to provide a quick evaluation of the joint effusion, cold abscess, or soft tissue masses. Computed tomography (CT) is helpful in the detection of bone involvement, periosteal reaction and calcifications, and soft tissue abscesses [11], making MRI the modality of choice to demonstrate the changes in the soft tissues and bone marrow in tubercular arthritis involving a single large or medium joint (Figures 2A, 2B, 2C) [12].

Sickle cell disease

**Figure 3:**
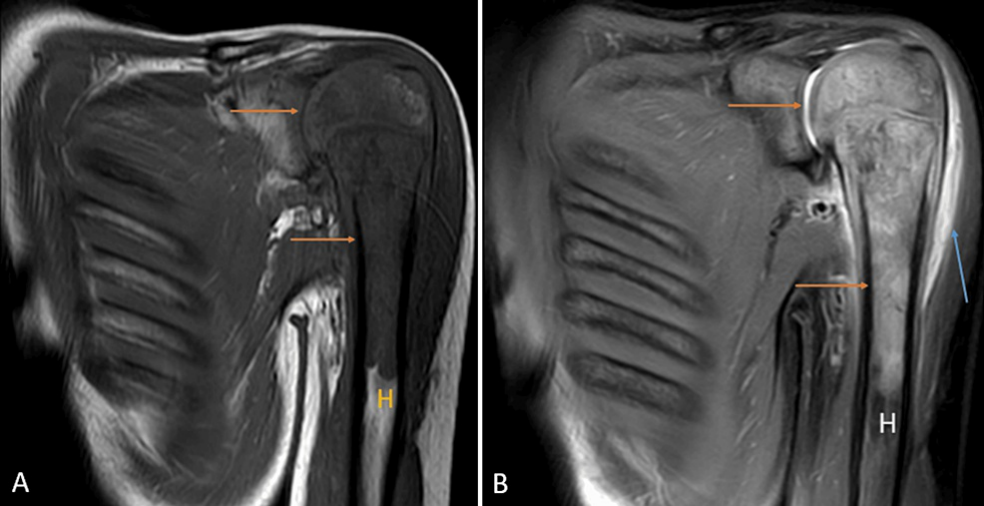
A seven-year-old male with sickle cell disease with marrow reconversion and myositis. Coronal T1 weighted imaging (A) shows a hypointense signal in the proximal shaft and neck of the humerus (orange arrows) with mild expansion of the involved areas. Coronal fat-suppressed proton density (B) shows hyperintense signals in the proximal shaft and neck of the humerus with mild expansion of the involved areas (orange arrows) suggesting bone marrow reconversion. Also, hyperintense signal is seen in the short head of the triceps suggesting myositis (blue arrow). H = humerus Image credits: Anshul Sood

Sickle cell disease (SCD) is a hereditary disorder of the red blood cells (RBC) and causes crescent-shaped distortion or sickling of the RBC. This abnormal shape of the RBC causes anemia, which advances to increased production of the RBCs, causing bone expansion, extramedullary blood cell formation, hematopoietic marrow hyperplasia, and pathologic fractures. Normally, the red marrow is converted into the yellow marrow with age. However, in patients with SCD, there is persistence of red marrow with reconversion of the yellow marrow, causing abnormal low signal intake on T1 weighted imaging. Ischemia and tissue hypoxia might lead to bone infarctions and, eventually, osteosclerosis, which give a 'bone within bone' appearance to the affected area. Fibrotic changes and sclerosis due to long-standing infarction give a hypointense signal on T1WI and T2WI. Relative areas of ischemia caused by reduced flow in the small blood vessels and associated immunocompromise due to hyposplenism act as a nidus for the development of osteomyelitis giving hyperintense signal on T2WI and hypointense signal on T1WI (Figures 3A, 3B) [13].

Metastasis

**Figure 4:**
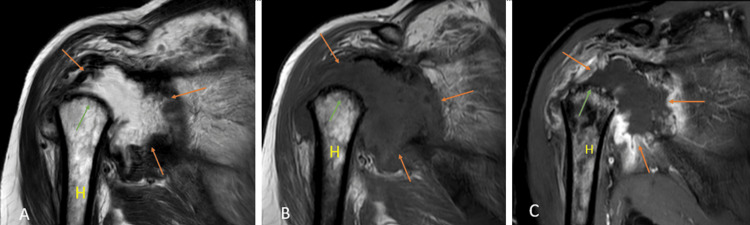
A 43-year-old female with lung cancer metastasis. Coronal T2 weighted imaging (A), T1 weighted imaging (B), and contrast-enhanced T1 weighted imaging (C) show peripherally enhancing collection (orange arrows) and destruction of neck and head of humerus {H}, and glenoid labrum (green arrow) with increased joint space. Image credits: Anshul Sood

On T1WI, osseous metastasis appears as a focus of low signal intensity. On T2WI, due to elevated water content, metastasis shows a hyperintense signal, and increased enhancement is seen post-gadolinium contrast enhancement because of increased vascularity [14]. In a study done by Thai DM et al. [15] on 93 patients with shoulder joint metastasis, it was found that carcinoma from various parts of the body can metastasize to the shoulder joint, breast carcinoma being the most common histotype, followed by myeloma, renal and lung cancers. Metastasis typically affects the proximal and middle one-third of the humerus, and distal metastasis is extremely rare (Figures 4A, 4B, 4C).

Rheumatoid arthritis

**Figure 5:**
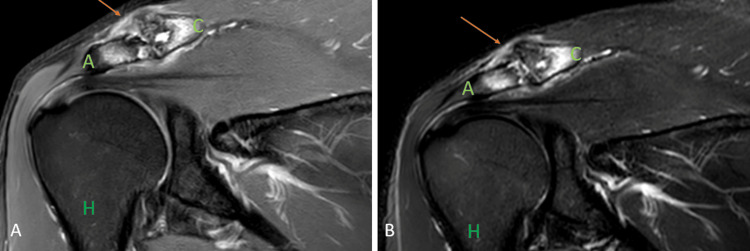
A 45-year-old male with rheumatoid arthritis of the acromioclavicular joint. Coronal fat-suppressed proton density sequence (A), and short tau inversion recovery sequence shows peri-articular erosion at the acromion {A - green color} and distal clavicular {C} joint with marrow edema and hyperintense signal in peri-articular soft tissues (arrow). H = humerus Image credits: Anshul Sood

Rheumatoid arthritis is a chronic systemic disease affecting the synovium. The earliest changes are non-osseous; hence, MRI is the first preference for imaging. The superolateral aspect of the head of the humerus is a classic location. The earliest features of the disease include hyperemia, synovitis, effusion, and bone marrow edema, which gives hyperintense signals on T2WI and shows enhancement post-contrast administration. Periarticular osteoporosis, joint space narrowing, and erosions leading to bone disfigurement and the destruction of the soft tissues occur as the disease progresses. A late finding includes a subgroup of the loose bodies known as rice bodies, which resemble polished rice and show iso-to-hypointense signals on T1WI and T2WI (Figures 5A, 5B) [16].

Soft tissue myxoma

**Figure 6:**
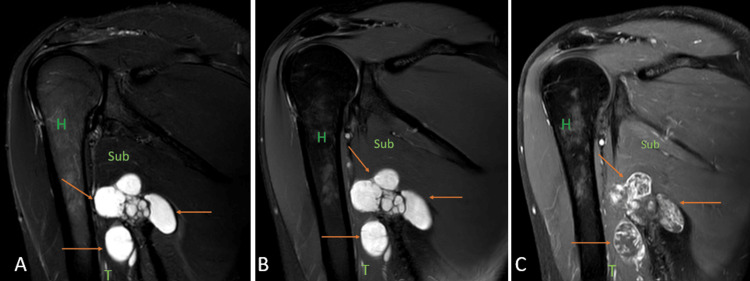
A 55-year-old male with soft tissue myxoma. Coronal short tau inversion recovery sequence (A), fat-suppressed proton density sequence (B), and contrast-enhanced T1 weighted imaging (C) show well-defined heterogeneously enhancing lobulated soft tissue mass lesion in the posterior aspect of the axilla (arrows) involving the triceps muscle {T} and abutting the subscapularis muscle {Sub}. H = humerus Image credits: Anshul Sood

Intramuscular myxomas are mesenchymal and are a benign variety of soft tissue myxomas believed to emerge from mucopolysaccharide-producing fibroblasts [17]. On MRI, they show a hypointense signal on T1WI, a hyperintense signal on T2WI, and a fat-suppressed proton density sequence. Post-contrast enhancement is variable and might show heterogeneous internal enhancement, circumferential and uneven internal enhancement, circumferential enhancement, or circumferential and linear internal enhancement (Figures 6A, 6B, 6C) [18].

Avascular necrosis

**Figure 7:**
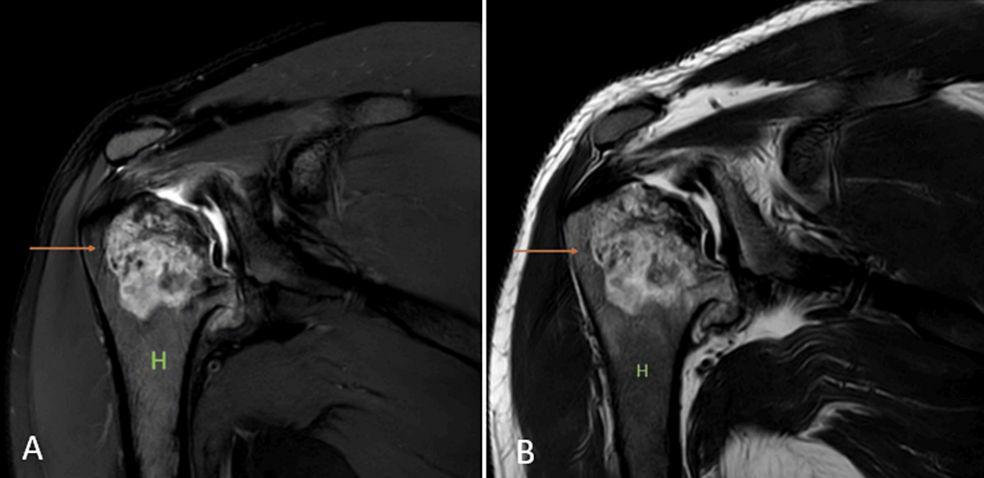
A 27-year-old male with avascular necrosis of shoulder joint. Coronal fat-suppressed proton density sequence (A) and coronal T2 weighted imaging (B) show geographical lesions with irregular margins with heterogenous signal intensity (arrow) involving the neck of the humerus {H} with erosion and destruction of the head of the humerus. Image credits: Anshul Sood

Interruption of the normal blood supply to the head of the humerus results in the death of cells in the bony matrix, which leads to resorption and remodeling of the affected bone, causing subchondral bone collapse. It leads to arthritic changes and joint incongruity [19]. CT may appreciate alterations in bone density but is insensitive to detecting early stages of avascular necrosis (AVN) [20]. MRI is extremely sensitive and specific in efficiently diagnosing the early changes of shoulder osteonecrosis and is the modality of choice for imaging in AVN. On T1WI, bands like hypointense signals are noted, which appear hyperintense on T2WI and fat-suppressed proton density sequence. Joint effusion and bone marrow edema may also be visualized (Figures 7A, 7B) [21].

Hill-Sachs, Perthes, and gleno labral articular disruption (GLAD)

Anterior stability to the shoulder joint is given by the anterior labroligamentous complex, which consists of the antero-inferior labrum and inferior glenohumeral ligament. Bankart lesion is the most common labral injury caused due to anterior instability. It leads to antero-inferior labrum avulsion and disorganization of the labroligamentous complex along with anterior scapular periosteum [22].

**Figure 8:**
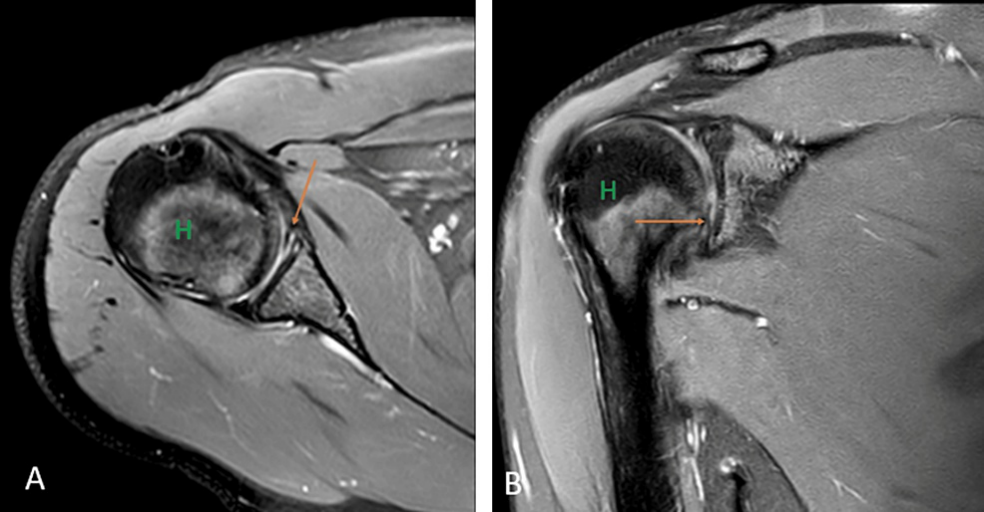
A 17-year-old male with Perthes. Axial (A) and coronal (B) fat-suppressed proton density images show detachment of the anteroinferior labrum with medially stripped but intact periosteum. H = humerus Image credits: Anshul Sood

Perthes and GLAD are types of Bankart lesions. Perthes consist of the avulsion of the antero-inferior part of the labrum with stripped but unmutilated scapular periosteum (Figures 8A, 8B).

**Figure 9:**
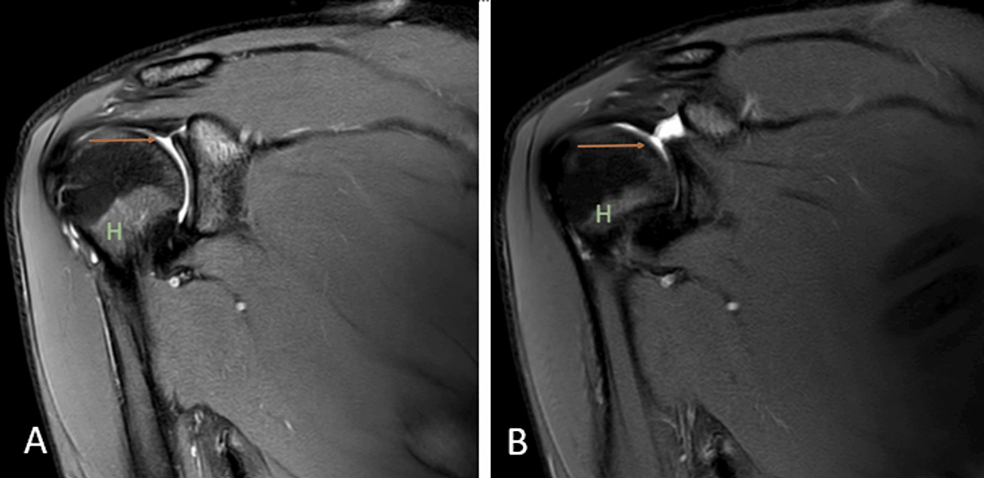
A 20-year-old male with posterior gleno-labral articular disruption (GLAD). Coronal fat-suppressed proton density (A) and (B) images show discontinuity in the glenoid labrum with hyperintensity in the defect (arrows). H = humerus Image credits: Anshul Sood

The lesion is best visualized in the abduction-external rotation (ABER) position. GLAD involves a superficial tear of the antero-inferior labrum with associated injury to the articular cartilage (Figures 9A, 9B) [22].

**Figure 10:**
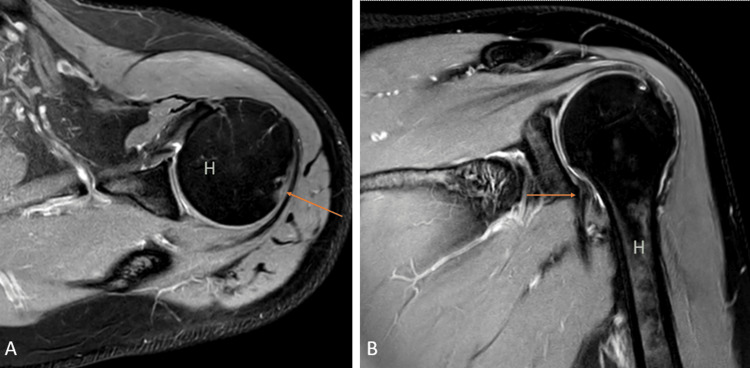
A 40-year-old male with a bony Hill-Sachs lesion. Axial fat-suppressed proton density (A) shows loss of normal circular shape of the postero-lateral surface of the head of humerus (arrow); and coronal fat-suppressed proton density (B) shows fraying of the anteroinferior glenoid labrum (arrow). H = humerus Image credits: Anshul Sood

Another imaging marker for anterior instability is the Hill-Sachs lesion, a defect in the posterosuperior region of the head of the humerus because of repeated anterior dislocation (Figures 10A, 10B) [22].

Partial articular supraspinatus tendon avulsion (PASTA)

**Figure 11:**
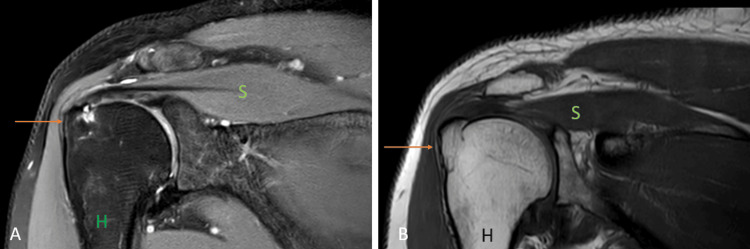
A 55-year-old male with partial articular surface supraspinatus tendon avulsion (PASTA). Coronal fat-suppressed proton density sequence (A) and coronal T1 weighted imaging (B) show the hyperintense signal at the greater tuberosity of the humerus {H} at the insertion of the supraspinatus muscle {S} with surface irregularity (arrow). Image credits: Anshul Sood

Partial articular-sided supraspinatus tendon avulsion (PASTA) lesion is a subset of partial thickness rotator cuff tear (Figures 11A, 11B) [23]. Overhead athletes, the younger population, and smokers are most commonly affected [24,25]. The pain is present mainly on the arc of motion ranging between 60˚ and 120˚ [26], and it is challenging to diagnose this pathology clinically because some patients are asymptomatic [27]. USG is considered a primary imaging technique because of its low cost, safety, and accuracy in detecting full-thickness rotator cuff tears [28]. However, for imaging partial thickness rotator cuff tears, MRI is more sensitive than USG and can detect subtle abnormalities involving the rotator cuff [29].

Avulsion fracture

**Figure 12:**
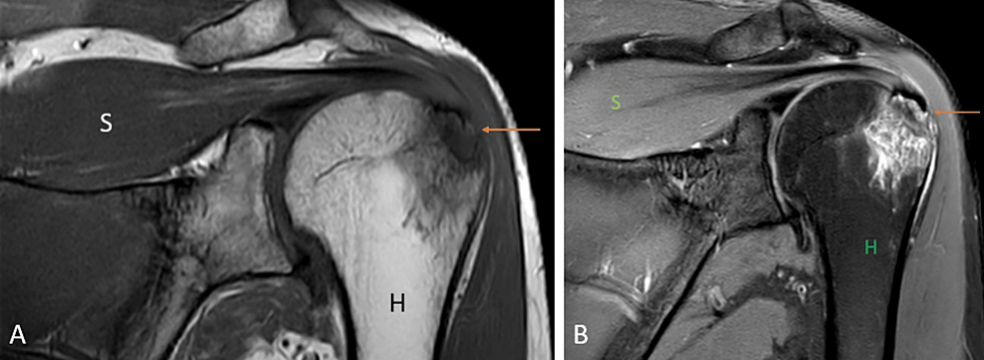
A 35-year-old male with an avulsion fracture of the supraspinatus tendon. Coronal T1 weighted imaging (A) and fat-suppressed proton density imaging (B) show avulsion fracture of the supraspinatus {S} tendon at its insertion at greater tuberosity of the humerus {H} which shows marrow edema (arrow). Image credits: Anshul Sood

Younger and healthier populations comprise most of the greater trochanter fractures [30]. Previous studies have shown that the severity of bone injury in the proximal humerus fractures correlates with the injury to the soft tissues [31]. CT demonstrates the bony injuries excellently; however, for the visualization of the extent of soft tissue injury, MRI is the modality of choice. MRI shows edema at the greater tuberosity with a hypointense line on T1WI, suggesting occult fracture (Figures 12A, 12B) [22].

Fracture malunion

**Figure 13:**
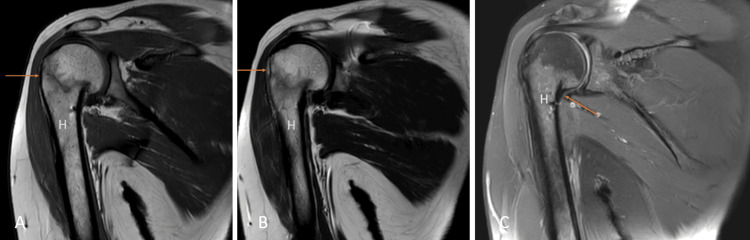
A 45-year-old female with a malunited fracture of the proximal humerus (neck). Coronal T1 weighted image (A), T2 weighted image (B), and fat-suppressed proton density image (C) show a malunited fracture of the proximal humerus (neck). Image credits: Anshul Sood

When the bone fails to heal in the normal anatomical position, it leads to the abnormal fixation of the bone, producing a rim of hypointense signal on T1WI and T2WI (Figures 13A, 13B, 13C). Malunion might occur due to various causes, including loss of reduction, inaccurate reduction, or inadequate mobilization of the fractured fragments. Post-traumatic osteoarthritis is a common complication, particularly in cases of intra-articular malunion [32].

Myositis

**Figure 14:**
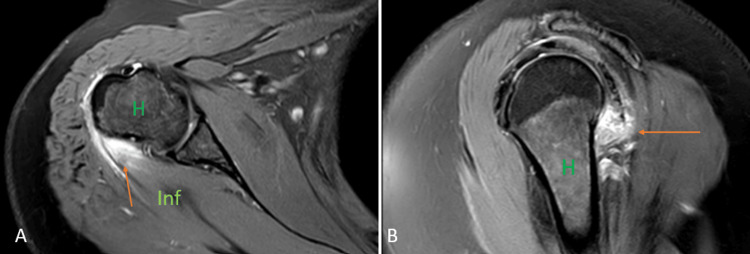
A 39-year-old female with infraspinatus myositis. Axial fat-suppressed proton density sequence (A) and sagittal fat-suppressed proton density sequence (B) show hyperintense signal in the infraspinatus muscle {Inf} at the greater tubercle. H = humerus Image credits: Anshul Sood

Myositis is a subtype of myopathy and refers to the inflammation of the muscles with an extensive list of etiologies, including inflammatory, infectious, traumatic, or drug-induced causes. MRI is the gold standard for non-invasive myositis assessment and gives hyperintense signals on T2WI and fat-suppressed proton density sequence due to edema (Figures 14A, 14B). On post-contrast administration, the contrast enhancement is appreciated in the inflamed muscles. With time, atrophy and fatty replacement of the affected muscles produce high-intensity signals on T1WI [33].

Repaired rotator cuff

**Figure 15:**
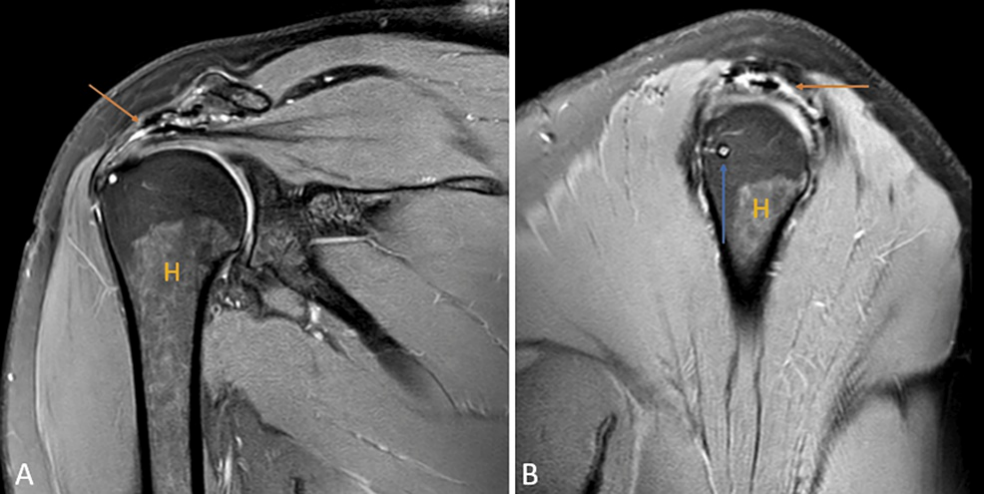
A 27-year-old male with a sprain of supraspinatus in an operated case of arthroscopic repair of supraspinatus. Coronal (A) and sagittal (B) fat-suppressed proton density images show a hyperintense signal at the insertion of the supraspinatus tendon (orange arrows) with implanted screw (blue arrow) noted in the anterosuperior part of the humerus {H}. Image credits: Anshul Sood

In a patient operated for rotator cuff muscle tear, it is not uncommon to visualize the same subset of muscle undergoing injury (Figures 15A, 15B). It is essential to understand that the repaired tendons commonly show an altered signal intensity in the first postoperative year. A proper history and clinical examination are crucial to correlate with MRI findings. Other causes of pain, like dislodgement of hardware, infection, or synovitis, can be picked up by MRI in addition to assessing the integrity of the rotator cuff and diagnosing re-tear [34].

Impingement syndrome

**Figure 16:**
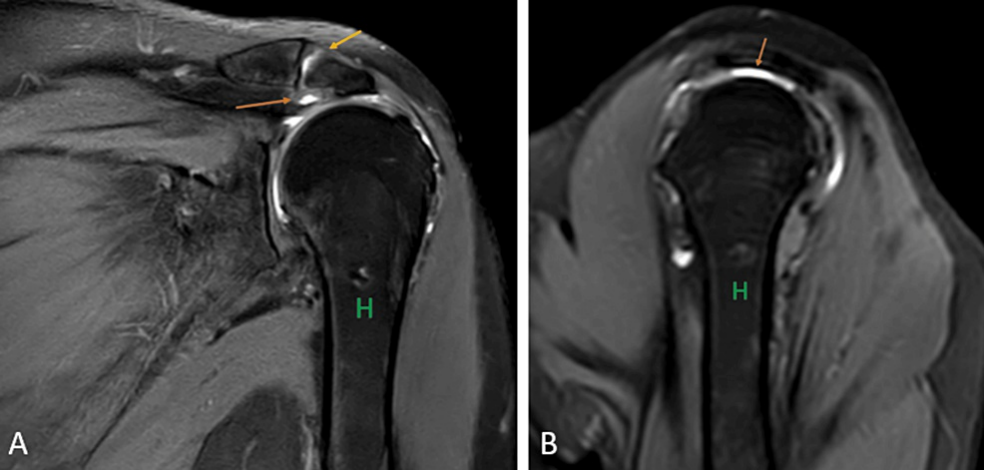
A 46-year-old male with supraspinatus tendinopathy due to external impingement. Coronal (A) and sagittal (B) fat-suppressed proton density sequence shows acromion impingement over the supraspinatus tendon with hyperintensity near its humeral attachment (orange arrow). Degenerative changes at the acromioclavicular joint in the form of a decrease in joint space and increased signal intensity (yellow arrow). H = humerus Image credits: Anshul Sood

Shoulder impingement (Figures 16A, 16B) is a group of conditions characterized by the entrapment of the musculoskeletal soft tissues within the shoulder and results in pain [35]. It has been classified into internal impingement, intrinsic (non-outlet) impingement, and primary and secondary extrinsic (outlet) impingement [36]. Subacromial impingement is the most prevalent form of the primary extrinsic impingement syndrome [37]. MRI is the imaging modality of choice because of its superior imaging quality of the soft tissues and the bony abnormalities like acromioclavicular joint capsular hypertrophy and bony spurs [38].

Rotator cuff tear

**Figure 17:**
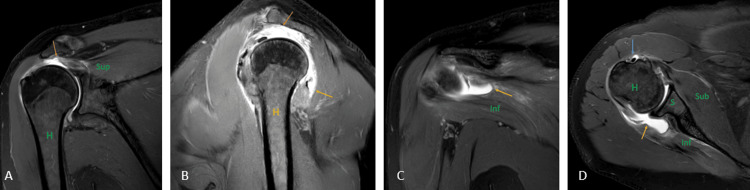
A 51-year-old male with a complete tear of the supraspinatus tendon, a partial tear of the infraspinatus tendon, and the bicipital tendinopathy. Coronal (A) and (C), sagittal (B), and axial (D) fat-suppressed proton density sequence show hyperintensity along the fibers of supraspinatus near its attachment to the humeral head with complete disruption of fibers suggesting complete tear (orange arrow); hyperintensity along the fibers of infraspinatus near its attachment to the humeral head with partial disruption of fibers suggesting partial tear (yellow arrow). Hyperintensity along the tendon of the long head of the biceps in the bicipital groove suggesting bicipital tendinopathy (blue arrow). H = humerus; S = scapula; Sub = subscapularis; Sup = supraspinatus; Inf = infraspinatus Image credits: Anshul Sood

Rotator cuff tears (Figures 17A, 17B, 17C, 17D) are one of the most common causes of pain in the shoulder joint and are mostly seen in the adult population. The prevalence of tears increases with age, and the etiology can be either traumatic or degenerative [39]. USG has a high specificity and sensitivity in diagnosing full-thickness rotator cuff tears but has many limitations, including the examiner's experience, and limited joint range of motion, especially external rotation [40]. It has been reported by a recent meta-analysis that on the present level of knowledge, MRI has the best sensitivity and specificity for detecting rotator cuff tears. Injuries to variants of insertion of pectoralis minor also mimic the rotator cuff tear which is inserted at the rotator interval of the glenoid capsule near the insertion of the supraspinatus tendon [29,41].

## Conclusions

The high frequency of patients with shoulder pain is mainly due to internal derangements of the shoulder joint. Various imaging modalities, like USG, CT, and MRI, evaluate these internal derangements. USG is the first imaging modality for shoulder joint pathologies because of its safety, low cost, and lesser time for evaluation; however, it is not very accurate in assessing all the soft tissue pathologies and is completely useless in assessing bone marrow changes. MRI is highly specific and sensitive in assessing muscles, joints, ligaments, bone, and other soft tissue structures, making it the imaging modality of choice. In this pictorial essay, we have discussed the MRI imaging features of various typical and atypical pathologies affecting the shoulder joint.
